# Thoracic and Abdominal Mesothelioma in an Older Horse in Lazio Region

**DOI:** 10.3390/ani12192560

**Published:** 2022-09-25

**Authors:** Giuseppe Passantino, Emilio Sassi, Ilaria Filippi, Valerio Serata, Antonella Tinelli, Nicola Zizzo

**Affiliations:** 1 Department of Veterinary Medicine, University of Bari “Aldo Moro”, Strada Prov.le per Casamassima Km.3 Valenzano, 70010 Bari, Italy; 2 Independent Researcher, Campagnano di Roma, 00063 Roma, Italy; 3 Independent Researcher, Genzano di Roma, 00045 Roma, Italy; 4 “Equine Practice srl”, Strada Valle del Baccano 80, Campagnano di Roma, 00063 Roma, Italy

**Keywords:** mesothelioma, aged horse, immunohistochemical labelling, thorax, abdomen, carcnoma

## Abstract

**Simple Summary:**

Mesothelioma in the equine species is poorly described in Italy. It is important to make an early and error-free diagnosis for a correct clinical approach, as well as to establish the triggering cause of this neoplasm. The etiology, most likely of an environmental nature, would reveal a valid biological indicator in the horse, far from urbanized centers.

**Abstract:**

A Quarter Horse, a gelding aged 22, was subjected to a clinical examination for colic syndrome. During admission to the clinic, blood counts and ultrasound examination were performed. Ultrasound revealed abdominal masses and abundant accumulation of pleural (50 L) and abdominal fluid (100 L). Cytology was performed on the aspirated fluid. The patient was euthanized. The autopsy examination revealed abundant effusion and nodular masses on the peritoneum, omentum, lungs, heart, and mediastinum. A diagnosis of epithelioid mesothelioma was made via histopathology and confirmed with immunohistochemistry; it showed positive antibodies against cytokeratin (CK) and vimentin. Mesothelioma is a rare cancer in older horses. It is important to employ the correct differential diagnostics using the available methods, providing valid ante-mortem support to the clinical veterinarian and monitoring the territory using this species as a valid biological indicator.

## 1. Introduction

According to Williams, in a necropsy study conducted by the Veterinary Diagnostic Laboratory of the University of Kentucky, malignancies occur in 8% of horses between the ages of 15 and 19, with a 17% increase in horses aged 30 and over [1]. The most frequently diagnosed tumors in older horses are pituitary adenoma, melanoma, squamous cell carcinoma, thyroid adenoma, sarcomas, and abdominal lipoma; multiple neoplasms are rare, except for multiple endocrine tumors, which can be associated with other pathologies of the thyroid gland, adrenal glands, and pituitary gland, in some respects resembling the human multiple endocrine neoplasia (MEN) syndrome [1,2,3,4,5]. Mesothelioma is a tumor that arises from the cells of the mesothelium (pleura, peritoneum, pericardium and vaginal tunica). In veterinary oncology [6] and according to the WHO, mesothelioma is classified into:

- **Epithelioid Mesothelioma**—the most common, with the best prognosis. It consists of small cells of an oval to cuboidal shape that connect each other, often forming small tubular or papillary structures;
- **Sarcomatoid or fibrous mesothelioma**—this is made up of elongated cells (fusiform). Contrary to the epithelioid-type mesothelioma, these cells do not stick together to form structures and spread widely into surrounding tissue;
- **Mixed or biphasic mesothelioma**—called biphasic, at least 10% of each cell type must be present in the tissue samples examined (epithelioid and sarcomatoid cells) [7].

The mesothelioma can affect different species, including dogs [8], cats [9], cattle [10], sheep [11], goats [12], and horses [2,13,14,15,16,17,18]. The onset in horses is very rare. Since 1976, there have been around 11 reports of mesothelioma in horses located in the pericardium, pleural cavity, peritoneal cavity, or in more than one of these organs. The majority of the reported cases involve females between the ages of 2 and 27, with a higher incidence in adults and the elderly patients. There is no obvious breed predisposition [19]. In most cases of equine mesothelioma, the diagnosis is made post-mortem at the slaughterhouse or during a clinical necropsy.

Evidence of severe lesions of the mesothelium in the thoracic and abdominal cavity, along with the presence of few publications of mesothelioma cases described in Italy, has led us to investigate this neoplastic process using the traditional histological technique, along with an immunohistochemical study. Our study has led us to hypothesize that horses, in the mentioned geographical area, could be considered excellent sentinel animals; in fact, considering the areas of environmental pollution between humans and animals, as reported by the Commission of the Academy of Science, animals were proposed as a sentinel system (sentinel species application, SSA) for various air pollutants, including asbestos, ascribing to animals a key role deemed to be essential for human epidemiological studies and showing the effects of those mineral fibers and their role in biological response [20,21,22,23].

## 2. Materials and Methods

### 2.1. Clinical History

We presented a case of a male gelding horse, a 22-year-old Quarter Horse with a chestnut coat, stabled in paddock and involved in equestrian tourism activities. The owner has not reported any pathological events in the previous 22 years. The horse was admitted to the equine veterinary clinic “Equine Practice s.r.l.” located in Campagnano di Roma (Rome), after a visit to the field, for colic pain and sensory depression. Upon admission, the horse presented a heart rate of 80 beats per minute (b.p.m.), 24 respirations per minute (r.p.m.), congested mucous membranes, hematocrit 60%, an absence of intestinal motility, and leukopenia of 4.000 white blood cells (wbc) (reference interval: 6.000–9.000).

**Figure 1:**
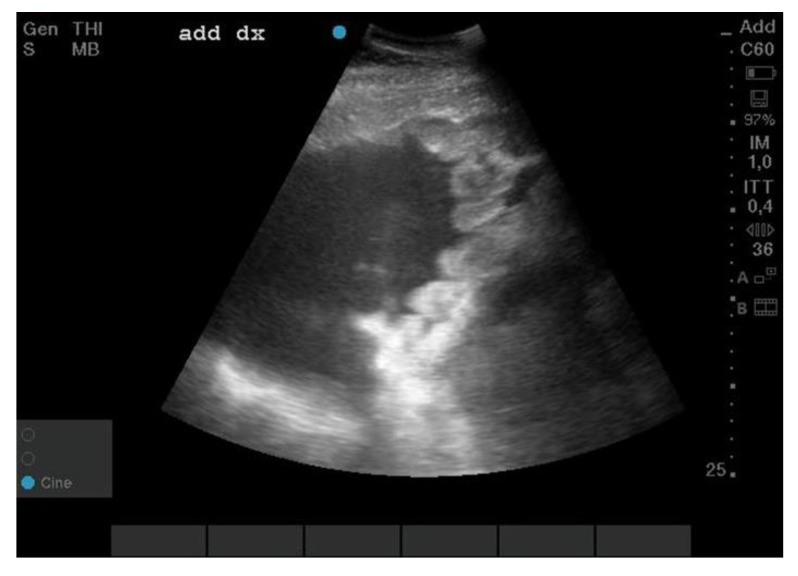
Abdominal ultrasound. Right middle abdomen window: presence of abundant amount of free hypoechoic fluid, and thickening of the parietal peritoneum where rounded formations adhere to clusters.

The abdominal ultrasound showed ascites with evidence of abundant free hypoechoic fluid in which the viscera were floating and diffuse and hyperechoic rounded formations were adhering to a reactive peritoneum (Figure 1); the thoracic ultrasound examination was unremarkable. During the hospitalization, the abdomen was drained through a ventral access with a rigid sterile cannula; the first drain produced about 100 L of serum-blood fluid, and the second drain produced about 60 L (1.200 wbc and 2 g/dl PT), for which the cytological examination was carried out.

The horse was treated with antibiotics (cephalosporins, marbofloxacin, and metronidazole), dexamethasone, fluid therapy and plasma.

After 5 days of hospitalization, given the poor response to therapy and the drastic worsening of clinical conditions, with clinical signs of hypovolemic shock, we opted for euthanasia for humanitarian purposes. After about two hours, the animal was subjected to an autopsy.

### 2.2. Cytological Examination

During the abdominal drainage (performed ante-mortem through ventral access with a sterile rigid cannula) fluid material (20–30 cc) was collected and, after fixation, prepared in a “thin layer” with the THINPrep method and stained with the May Grünwald–Giemsa method (MGG).

### 2.3. Histological and Immunohistochemical Examination

The samples were fixed in 10% buffered formalin, processed with Histokinette 2000 (Reichert-Jung Gmbh, Nussloch, Germany) and embedded in paraffin. The inclusions were microtome-cut, and serial sections of 4 µm were obtained, placed on a slide and stained with Hematoxylin-Eosin (H. & E., Bio-Optica, Milan, Italy), Masson’s trichrome (Bio-Optica, Milan, Italy), and periodic-Schiff acid (PAS) (Bio-Optica, Milan, Italy). Immunohistochemistry was performed according to the streptavidin-biotin (LSAB) method (Agilent-Dako, Glostrup, Denmark) on paraffin sections. The tissue sections (4 μm thick) were placed on poly-L-lysine coated slides, subsequently deparaffinized in xylene, dehydrated in 95% ethanol, and rehydrated in distilled water. The antigenic unmasking was carried out with citrate buffer (0.1, pH 6) and placed for 15 min in microwave. All sections were treated for 30 min with 0.3% hydrogen peroxide, then in methanol for 12 min to quench the endogenous peroxidase activity (12 mL of H_2_O_2_ in 400 mL of methanol). After three 5-min washes in phosphate buffered saline (PBS), the sections were blocked by soaking for 20 min at room temperature in PBS containing 1% bovine serum albumin. They were subsequently incubated overnight at 48 °C with primary antibodies vimentin, cytokeratin (CK) AE1/AE3, CK 5/6, and CK 8/18 (Agilent-Dako, Glostrup, Denmark) (Table 1). After washing carefully with Tris saline solution (pH 7.6), the sections were first incubated with the secondary antibody (anti-rabbit IgG conjugated with biotin) for 30 min and then with streptavidin-peroxidase for 40 min. 3,3-diaminobenzidine (DAB) (Agilent-Dako, Glostrup, Denmark) was used as a chromogen. Gill’s hematoxylin (Polysciences, Warrington, PA, USA) was used to counteract the nucleus and then dehydrated and whipped. Positive controls were inserted for each antibody type during the immunohistochemical procedure using previously tested organ tissue samples as suggested by the data sheet. For the negative control, the primary antibody was omitted during immunohistochemical staining. The morphological interpretation of the immunohistochemical stains was performed independently by two of the authors (NZ and GP). The case was considered positive for a given marker only when both observers agreed on its specificity and distribution. Sections were initially examined with a magnification, per field, of ×200 (objective × 20 and eyepiece lens × 10; 0.7386 mm^2^ per field) and subsequently at ×400 (objective × 40 and eyepiece lens × 10; 0, 1885 mm^2^ for the field) using an optical microscope (Leica DM4000 B, Milan, Italy) equipped with a digital color camera and for analysis was performed using an interactive image analysis system (Alexasoft x-plus). The results were interpreted as follows: negative reaction, foci of positivity “+” (<30% of positive cells), “++” (30–60% of positive cells), and “+++” diffuse positivity (>60%).

## 3. Results

### 3.1. Necroscopic Examination

**Figure 2:**
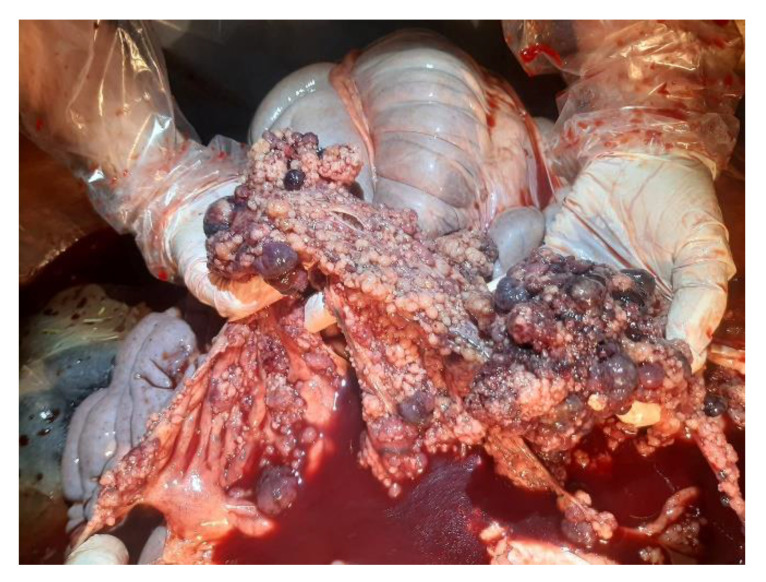
Abdominal cavity: dark red effusion and nodular masses, arranged in clusters.

**Figure 3:**
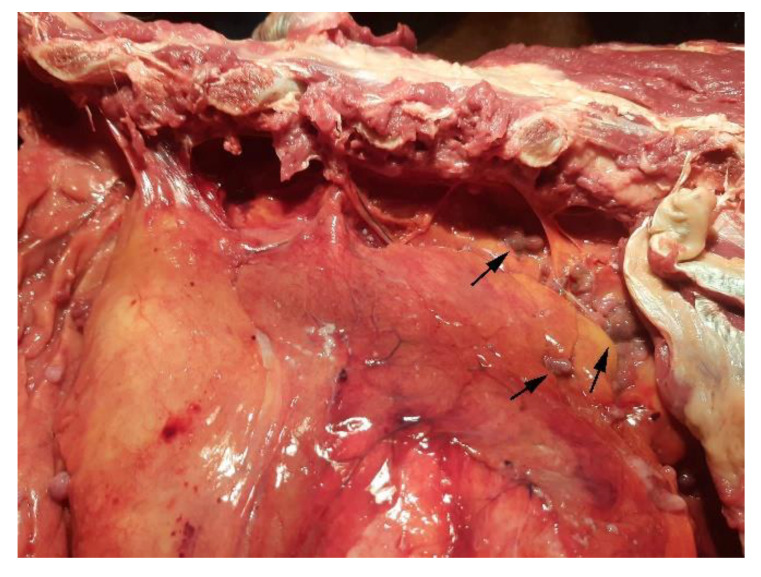
Lung: neoplastic nodules (arrows) and adhesions between the pleura and the chest wall.

**Figure 4:**
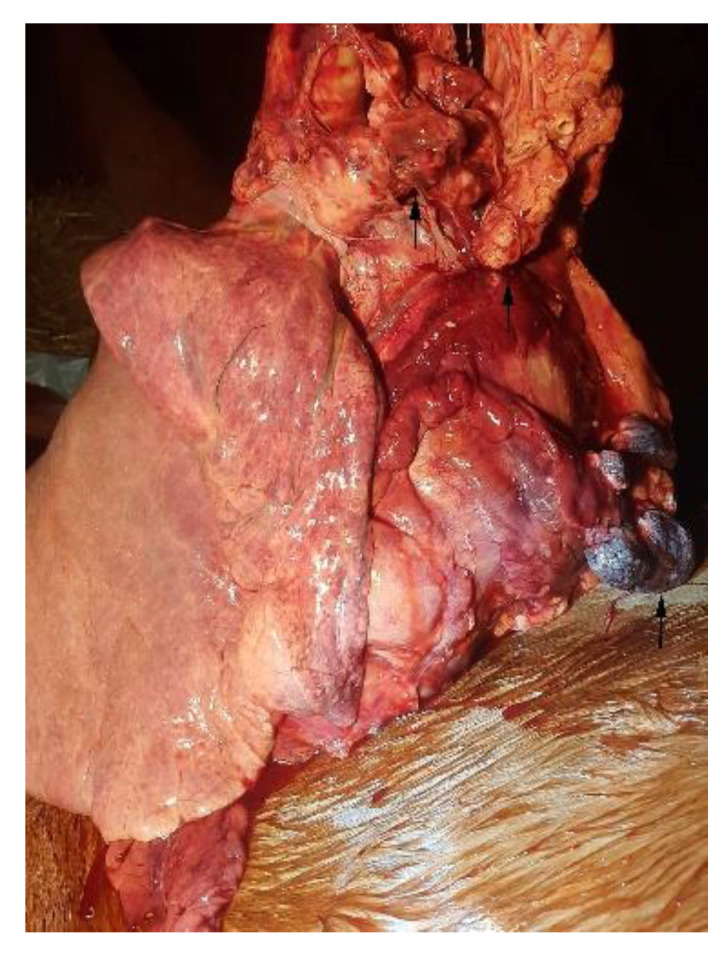
Lung: neoplastic nodules (arrows) arranged in clusters on the mediastinum.

**Figure 5:**
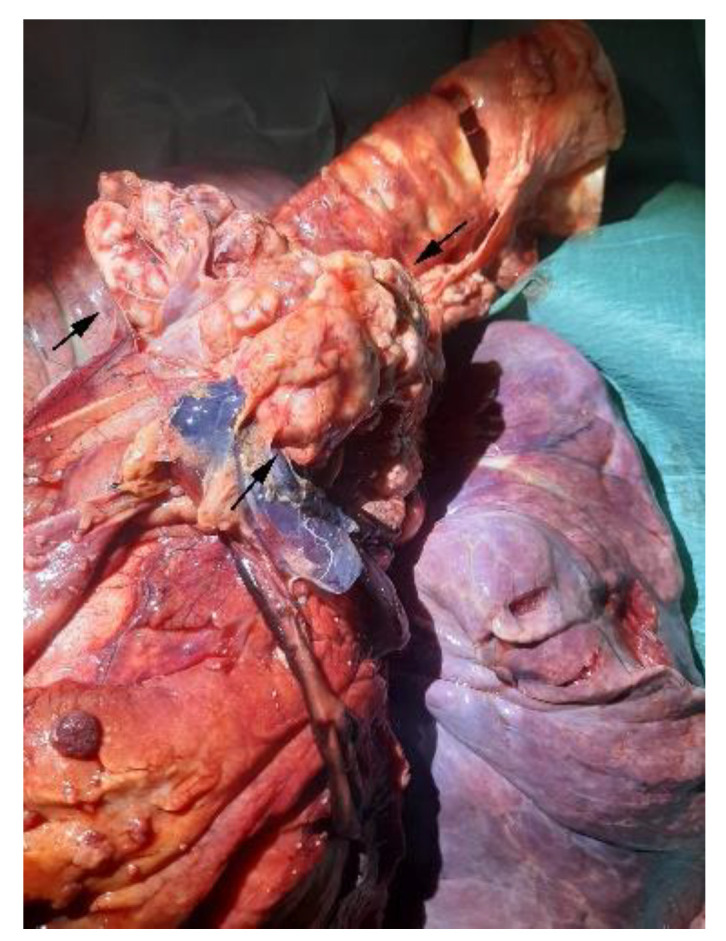
Heart: neoplastic mass at the base of the heart (arrows).

On external examination, the subject appeared emaciated with normal musculoskeletal development, congested mucous membranes, and an abdominal region that appeared enlarged and distended. Upon opening the abdominal cavity, the presence of a dark red ascitic fluid was observed, approx. 100 L; the topography of the abdominal organs was preserved and did not show alterations on the external surface and on the cut surface. The peritoneum and omentum had numerous nodular masses, up to 4 cm in size, diffuse, whitish and red, with a fatty appearance, and arranged in clusters (Figure 2). The vessels were ectasic. At the opening of the thoracic cavity, the presence of abundant liquid of about 50 L was observed; additionally, numerous adhesions between the pulmonary pleura and costal wall were observed (Figure 3). Rounded formations with a dark red color, varying in size from 0.5 cm to 2 cm, were scattered on the main lobes; the pericardial sac; and the base of the heart, the aorta, and the mediastinum (Figure 4 and Figure 5).

Considering the clinical findings, the neoplastic masses, and the abundant abdominal and pleural effusions, the main differential diagnoses include mesothelioma, carcinoma, carcinomatosis, and metastatic neoplasms.

### 3.2. Cytological Aspects

The smears showed mesothelial cells arranged in small clusters, intermingled with numerous scattered neutrophils and erythrocytes. Hyperchromatic and anisocytic tumor cells showed large irregular nuclei, coarse chromatin, and visible nucleoli. Basophilic cytoplasm is abundant and partly microvacuolized; cell margins were well defined or crinkled.

### 3.3. Histopathological Aspects

**Figure 6:**
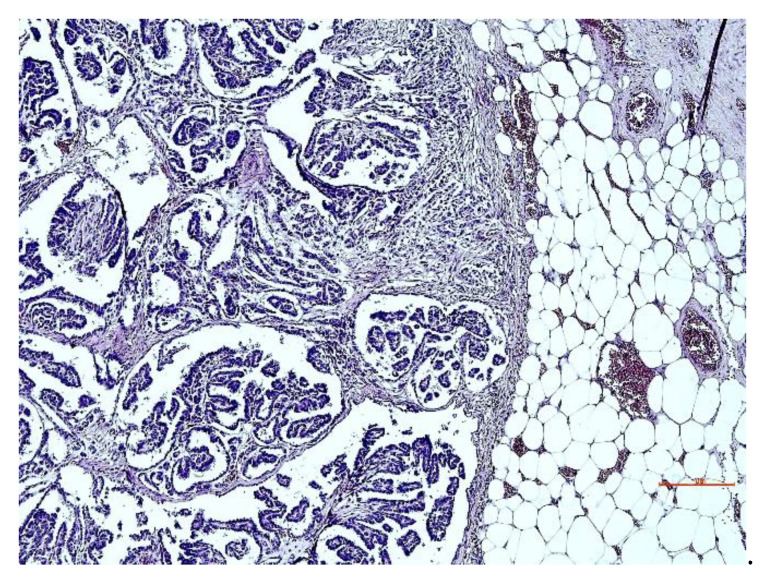
Lung: tubulo-papillary structure, with multiple layers of neoplastic cells. H & E stain. Bar = 100 µm.

The serosa of the nodular neoformations founded in the thoracic cavity; the pericardial sac; and the base of the aorta and the mesentery, supported by branched trabecular connective tissue (Masson’s trichrome stain positive) covered by one or more layers of neoplastic cells with marked anisocytosis and anisocariosis, of a columnar or cubic shape (Figure 6), often associated with extensive lymphoplasmacytic infiltration.

**Figure 7:**
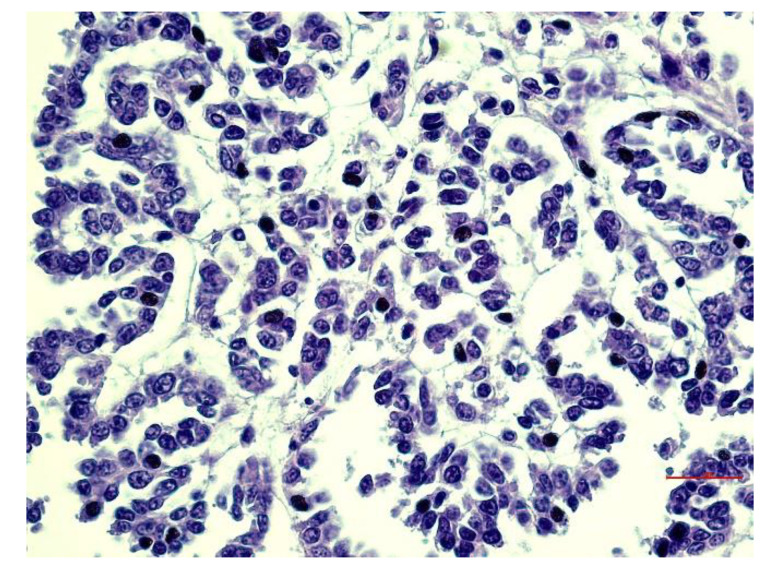
Lung: Hyperchromatic and ovoid nuclei in a subapical position with distinct nuclear membranes. Coarse chromatin and multiple nucleoli. H & E stain. Bar = 30 µm.

The hyperchromatic nuclei, located in the sub-apical position, were ovoid, bulky, and occasionally vacuolized with distinct nuclear membranes. Chromatin varies from a fine network to a coarse network, according to the degree of differentiation with multiple and well evident nucleoli. Mitoses were rare (Figure 7). PAS-positive eosinophilic cytoplasm was moderately abundant with indistinct cell boundaries. The final diagnosis corresponds to epithelioid mesothelioma with a tubulo-papillary structure. All observed neoplasms exhibited non-invasive behavior.

Vascular metastases consisted of epithelioid cells. The abdominal and tracheobronchial lymph nodes had a hemorrhagic parenchyma with paracortical and cortico-medullary sinuses infiltrated by neoplastic cells. No other organs were affected with neoplasm; the liver presented with severe steatosis, and the spleen appeared congested.

### 3.4. Immunohistochemical Aspects

**Figure 8:**
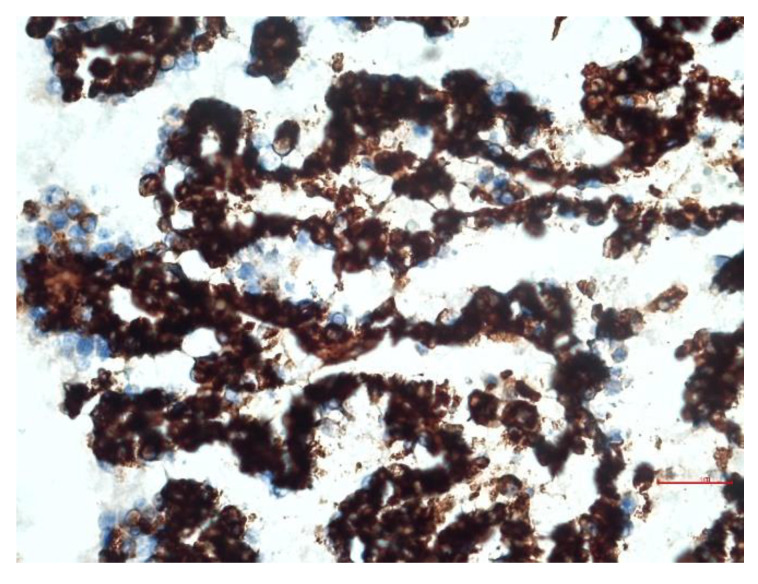
Lung: IHC vimentin: broad positivity of most cancer cells. Bar = 30 µm.

**Figure 9:**
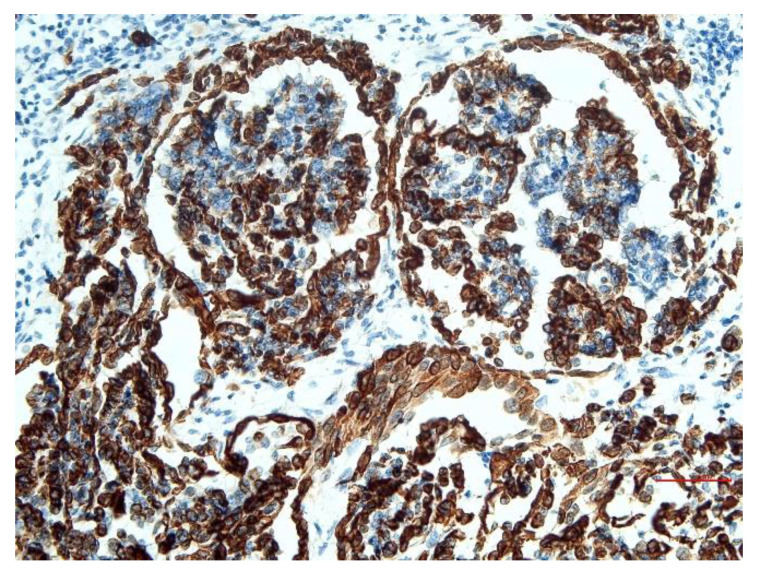
Lung: IHC cytokeratin: positivity over 75% of cancer cells. Bar = 60 µm.

Immunohistochemically, mesothelial cells showed cytoplasmic positivity at vimentin (Figure 8) and CK AE1/AE3, and cytoplasmatic and cellular membran positivity at CK 8/18 (Figure 9), all with positivity greater than 75%. The CK 5/6 affect less than 25% of cells.

## 4. Discussion

There are rare cases of peritoneal, pleural, and pericardial mesothelioma in the same patient, especially in elderly animals. They have been described in cats [24], but in horses there are no reports of this in the recent literature; the simultaneous involvement of the peritoneal and thoracic cavity has occasionally been reported in other types of equine mesotheliomas [13,17,25].

In the reported case, we hypothesized that the primary tumor that most likely originated from the peritoneum had metastasized to the local lymph nodes and chest cavity. However, a multicenter origin of the tumor cannot be completely ruled out. The noticeable collection of fluid in the abdominal and thoracic cavity of this horse could be secondary to the reduced drainage of the normally produced fluid, secondary to lymphatic and lymph node metastases. Alternatively, as hypothesized by Stoica et al., 2004 [17], the excessive production of fluid could have been secondary to the neoplastic mesothelial process.

Based on the clinical signs, colic syndrome, gastritis, and parasitism were all possible differential diagnosis, which were subsequently excluded via the performed diagnostics tests. Considering the necroscopic findings, the most probable differentials for peritoneal and thoracic masses included mesothelioma, carcinoma, carcinomatosis, and metastatic neoplasms.

Considering the histological subtypes of mesothelioma and their similarity with other neoplasms, the histopathological diagnostic challenge is represented in the correct diagnosis. In fact, the differential diagnosis for epithelioid mesothelioma includes carcinomas and epithelioid cancers; the differential diagnosis for sarcomatoid mesothelioma includes sarcomas and other spindle cell neoplasms; and the differential diagnosis of mixed mesothelioma comprises synovial sarcoma and metastatic pleomorphic carcinoma [7].

In this case, the neoplastic cells were characteristic of the epithelioid subtype. In humans, this mesothelioma is classified into further subtypes (papillary, tubular, cystic, and solid), and some of these are also used in dogs [6]. Based on this classification, the predominant pattern was the tubulo-papillary.

Immunohistochemistry is useful in order to distinguish mesothelioma from epithelial neoplasms. Mesothelial neoplastic cells are positive for both cytokeratin and vimentin, contrary to carcinomas, which are generally positive for cytokeratin and negative for vimentin [6,19]. In the reported case, the positivity of both immunohistochemical stains was useful in confirming the mesothelial origin of the neoplastic cells.

The cytological examination was carried out on the effusion taken before the autopsy. The greatest difficulty is to distinguish reactive mesothelial cells from neoplastic ones; in fact, the cytomorphological characteristics of reactive cells often overlap with the neoplastic ones, making it impossible to distinguish them from the cytological examination alone [26]. For these reasons, in order to correctly differentiate between mesothelioma carcinoma and adenocarcinoma, the use of immunohistochemistry and/or ultrastructural examination with an electron microscope becomes of paramount importance [27,28,29,30].

The aetiology of this tumor in this species is unknown; in humans and dogs, it has been reported that exposure to absestos dust induces the onset of mesothelioma [31]. The clinical signs shown correlate closely with the location of the tumor. Therapy is usually symptomatic and aimed at alleviating the animal’s suffering. Metastases are rare even if in this case they have been found in the pulmonary lymph node.

Sentinel animals offer, through the study of biological and health effects, an integrative approach in estimating the risk for human health. They allow one to highlight the presence of environmental contaminants, investigate their biodiversity in different environmental areas, and better estimate the risk deriving from contaminant exposure. Some animal species tend to spend their entire life in the same environment; their induction/latency time in developing a disease is shorter compared to human species; their diet and lifestyles are often unvaried; and medical treatments can be easily identified. In the geographical area reported in this paper, horse breeding is very developed, justifying our idea of using horses as a sentinel in detecting mesothelioma.

According to Knottenbelt, treatment would be impossible due to the rapid progression (4–6 weeks) resulting in euthanasia [19]. Fry et al. [26] performed an ante mortem diagnosis followed, however, by euthanasia.

Ours is a rare case of mesothelioma in the abdominal and thoracic cavity in a horse. To date, accurate ante-mortem ultrasound investigations showing peritoneal nodules of various sizes scattered on the serosa, along with laparoscopy, fine-needle aspiration, ultrasound-guided biopsy, and blood tests (haematology and biochemistry), can be of value, even if the ante-mortem diagnosis is difficult. In human medicine, studies have been carried out aimed at detecting particles of absestos from the fluids produced in excess and collected with fine-needle aspiration [32,33]. Considering the abundant effusion in the cavities, it is important to distinguish colic syndrome from mesothelioma in life, which is why this neoplasm often does not reach the pathologist’s observation. The collection of environmental data is also important. In fact, this case refers to a horse that lived in an area with a very high incidence rate of human mesothelioma (the National Mesothelioma Registry—Lazio). Another case came from the same area, in which both the horse and the owner were suffering from lymphoma.

## 5. Conclusions

Due to the environmental origin of this neoplasia, it is in our interest to monitor the other horses present in the same area in order to evaluate the presence of this neoplasia and propose the horses and the biological indicator of the disease. It would also be interesting to provide useful tools for an early diagnosis of the disease using similar diagnostic methods to those used in humans [32,33] and to propose an etiology for the neoplasm in this species that, to date, is still unknown.

Finally, this case highlights the diagnostic challenges encountered by the veterinary clinician and the pathologist in performing a diagnosis ante and postmortem in subjects suffering from mesothelioma.

## Figures and Tables

**Table 1:** The immunohistochemical markers used.

Antibody	Clone	Dilution	Company
CK 8/18 m	EP17/EP30	1/100	Dako, Glostrup, Denmark
CK AE1/AE3 m	AE1/AE3	1/100	Dako, Glostrup, Denmark
CK 5/6 m	D5/16 B4	1/100	Dako, Glostrup, Denmark
Vimentin m	V9	1/100	Dako, Glostrup, Denmark

## Data Availability

All data generated or analyzed during this study are included in this published article. If any additional material used and/or analyzed during the current study is required, it is available from the corresponding author on reasonable request.
